# *De novo *genome sequence assembly of a filamentous fungus using Sanger, 454 and Illumina sequence data

**DOI:** 10.1186/gb-2009-10-9-r94

**Published:** 2009-09-11

**Authors:** Scott DiGuistini, Nancy Y Liao, Darren Platt, Gordon Robertson, Michael Seidel, Simon K Chan, T Roderick Docking, Inanc Birol, Robert A Holt, Martin Hirst, Elaine Mardis, Marco A Marra, Richard C Hamelin, Jörg Bohlmann, Colette Breuil, Steven JM Jones

**Affiliations:** 1 Department of Wood Science, University of British Columbia, Vancouver, BC, V6T 1Z4, Canada; 2 BC Cancer Agency Genome Sciences Centre, Vancouver, BC, V5Z 4E6, Canada; 3 Amyris Biotechnologies, Inc., Hollis Street, Emeryville, CA 94608, USA; 4 Washington University School of Medicine, Forest Park Ave, St Louis, MO 63108, USA; 5 Natural Resources Canada, rue du PEPS, Ste-Foy, Quebec, G1V 4C7, Canada; 6 Michael Smith Laboratories, University of British Columbia, Vancouver, BC, V6T 1Z3, Canada

## Abstract

Sequencing-by-synthesis technologies can reduce the cost of generating *de novo *genome assemblies. We report a method for assembling draft genome sequences of eukaryotic organisms that integrates sequence information from different sources, and demonstrate its effectiveness by assembling an approximately 32.5 Mb draft genome sequence for the forest pathogen *Grosmannia clavigera*, an ascomycete fungus. We also developed a method for assessing draft assemblies using Illumina paired end read data and demonstrate how we are using it to guide future sequence finishing. Our results demonstrate that eukaryotic genome sequences can be accurately assembled by combining Illumina, 454 and Sanger sequence data.

## Abstract 2

A method for de novo assembly of a eukaryotic genome using Illumina, 454 and Sanger generated sequence data

## Background

The efficiency of *de novo *genome sequence assembly processes depends heavily on the length, fold-coverage and per-base accuracy of the sequence data. Despite substantial improvements in the quality, speed and cost of Sanger sequencing, generating a high quality draft *de novo *genome sequence for a eukaryotic genome remains expensive. New sequencing-by-synthesis systems from Roche (454), Illumina (Genome Analyzer) and ABI (SOLiD) offer greatly reduced per-base sequencing costs. While they are attractive for generating *de novo *sequence assemblies for eukaryotes, these technologies add several complicating factors: they generate short (typically 450 bp for 454; 50 to 100 bp for Illumina and SOLiD) reads that cannot resolve low complexity sequence regions or distributed repetitive elements; they have system-specific error models; and they can have higher base-calling error rates. To this point, then, *de novo *assemblies that use either 454 data alone, or that combine 454 with Sanger data in a 'hybrid' approach, have been reported only for prokaryote genomes, and no *de novo *assemblies that use Illumina reads, either alone or in combination with Sanger and 454 read data, have been reported for a eukaryotic genome.

In principle, it should be possible to generate a *de novo *genome sequence for a eukaryotic genome by combining sequence information from different technologies. However, the new sequencing technologies are evolving rapidly, and no comprehensive bioinformatic system has been developed for optimizing such an approach. Such a system should flexibly integrate read data from different sequencing platforms while addressing sequencing depth, read quality and error models. Read quality and error models raise two challenges. First, while it is desirable to identify a subset of high quality reads prior to genome assembly, and established read quality scoring methods exist for Sanger sequence data, there are no rigorous equivalents for 454 or Illumina reads [1]. Second, error models differ between different sequencing technologies.

A number of genome assemblers are currently available for combining Sanger and 454 read collections, as well as specialized short read assembly programs like ALLPATHS, SSAKE, Velvet and ABySS [2-5]. However, short reads require greater sequencing depth to ensure specificity in read overlaps, as shorter overlaps cause ambiguities in the assembly stage. This increased sequence depth prevents both applying the traditional overlap-layout-consensus method directly and extending Sanger/454 hybrid assemblers to use ultra-short reads. Assemblers that are primarily intended for short reads can process deep coverage read data; however, because read length and software limitations restrict the unambiguous sequence regions that they can assemble and they currently lack the capacity for scaffolding contigs effectively, they are typically limited to ultra-short reads. When we assessed such assemblers, the above challenges - likely compounded by the high error rate in our earlier Illumina read collections - resulted in contigs that were either too short or too unreliable to support comparing homologous blocks of sequence between genomes.

The Forge genome assembler [6] was designed for assembling combinations of reads from Sanger and 'next-generation' sequencing technologies, and attempts to address the above challenges. Distributed memory hash tables and pruned overlap graphs allow its classical overlap-layout-consensus approach to handle large data sets with deep coverage. Simulation techniques embedded in the algorithm allow it to automatically adapt to varying read lengths and error characteristics to accommodate rapidly changing performance in next-generation sequencing platforms.

In the work described here, we developed a hybrid approach that uses Forge for generating *de novo *draft genome sequences, and applied the approach to a filamentous fungus, *Grosmannia clavigera *(*Gc*). To generate the draft sequence, we combined: conventional, 40-kb fosmid paired-end (PE) Sanger reads from an ABI 3730xl sequencer; single-end (SE) 454 reads from Roche GS20 and GS-FLX sequencers; and PE reads from an Illumina Genome Analyzer (GA_ii_) sequencer. The current sequence assembly is approximately 32.5 Mb in length and has an N50 scaffold size of approximately 782 kb. The assembly as well as the raw read data are available from National Center for Biotechnology Information (NCBI; see Materials and methods). We describe how we prepared read data for assembly by filtering and trimming using an internally developed pipeline, which we make available [7]. We outline below our experience in assembling this eukaryotic genome using the Velvet and Forge assemblers. We also describe a bioinformatic approach for assessing the accuracy of such hybrid assemblies when no high quality reference sequence exists.

## Results

### Generating sequence data

**Figure 1:**
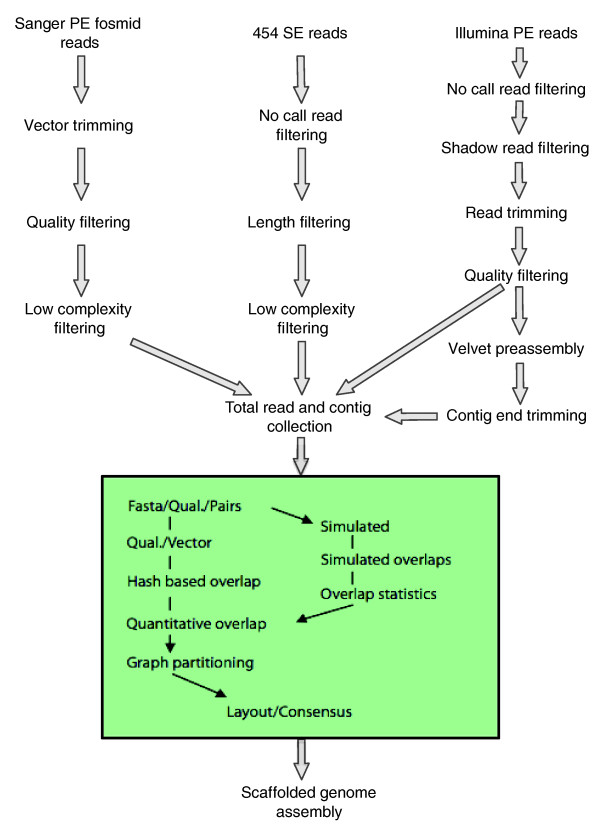
Assembly process overview. Overview of the process for producing *de novo *assemblies.

We assembled a genome sequence for *Gc *using the pipeline described below and in Figure 1. We first constructed a fosmid library, from which we generated 18,424 Sanger PE sequences (approximately 0.3-fold genome sequence coverage). We then used sheared genomic DNA to generate seven read sets on Roche GS20 and GS-FLX sequencers, producing 3,045,953 reads with 100.0 and 224.5 bp average lengths, respectively (250 Mb of sequence data; approximately 7.7-fold genome sequence coverage). Finally, we supplemented these data sets with PE, 42-bp reads (82,655,316) for a single library of approximately 200-bp sheared genomic DNA fragments on an Illumina GA_ii _(approximately 3.3 Gb of sequence data; approximately 100-fold genome sequence coverage).

### Initial assembly analysis

Initially, Illumina PE read data required preassembly, as we were unable to complete a Forge (v.20090319) run using our entire read collection; we integrated these data by preassembling them with Velvet. We assembled the read data described above, alone or in combination, and devised a strategy for refining these assemblies. Using Velvet (v.6.04 and v.7.31), we assessed assemblies generated from Illumina PE read data and Illumina with Sanger PE read data (see Materials and methods: Assembling Illumina data); using Forge we assessed assemblies generated from 454 SE read data, 454 SE with Sanger PE read data and 454 SE and Sanger PE read data plus a Velvet-preassembled contig backbone. We used a collection of 7,169 unique expressed sequence tag (EST) sequences to do an initial assessment of these assemblies. From the EST-to-genome alignments, we determined the number of complete alignments as well as the number of times an alignment was split between contigs in a resolvable ('partial') or unresolvable ('misassembly') manner (described in Materials and methods), and also identified small insertions or deletions (termed indels). The Velvet assembly generated from Illumina PE data alone yielded an N50 contig length of approximately 24.5 kb, and covered approximately 26.7 Mb of the 32.5 Mb manually finished genome sequence (Table 1). In contrast, a Forge assembly of the 454 read collection yielded an N50 contig length of approximately 7.8 kb and covered approximately 29.5 Mb of the complete genome sequence (Table 2). We checked the overlap between these assemblies, and found that 100% of the Velvet-Illumina assembly was contained within the Forge-454 assembly, while the 454 assembly contained an additional approximately 2.5 Mb of sequence that was not found in the Illumina assembly.

**Table 1:** Velvet assemblies

**ID**	**T42**	**T38**	**T36**	**T36; QRL(Q10) = 28**
Total contigs	6,945	8,637	19,118	39,488
N50 contig	24,566 (N/A)	10,706	2,902	1,299
Total DNA (bp)	26,721,397	26,466,756	25,854,719	24,812,690
EST analysis*	6,585/29	6,204/24	4,657/11	2,923/9

*EST alignments are given as: Complete alignments/Misassemblies (see Materials and methods). Velvet assemblies were generated from Illumina GA_ii _read data. Assembly T42 was generated from the untrimmed, no-call and shadow filtered Illumina PE reads. Assemblies T38 and T36 were generated by trimming the last 4 and 6 bp, respectively, from the T42 read set. Assembly T36, QRL(Q10) = 28 was generated with the T36 read set from which reads were removed if they failed the QRL(Q10) = 28 quality region length filtering (see Materials and methods).

**Table 2:** Forge assemblies

**ID**	**454**	**Sanger-454**	**Sanger-454-IlluminaPA**	**Sanger-454-IlluminaDA**
Total scaffolds*	7,860	4,805	2,307	1,443
N50 contig (scaffold)	5,773 (N/A)	7,440 (289,760)	31,821 (557,565)	164,278 (187,326)
Total DNA (bp)^†^	29,484,877	34,841,371	39,238,044	29,522,629
Number of scaffolds with gaps^‡^	0	656	163	17
Augustus predictions	10,555	10,230	8,912	8,476
EST analysis^§^	5,544/25	5,747/60	6,314/40	6,685/33

*Scaffolds included in this calculation contained two or more reads and were longer than 500 bp. ^†^Total DNA was calculated excluding gaps and was performed on scaffolds that contained two or more reads and were longer than 500 bp. ^‡^Gaps included in this calculation were longer than 50 bp. ^§^EST alignments are given as: Complete alignments/Misassemblies (see Materials and methods). Forge assemblies were generated using Illumina, 454 and Sanger read data. The '454' assembly was generated using only 454 SE read data. The 'Sanger-454' assembly was generated by combining the Sanger PE and 454 SE read collections. The 'Sanger-454-IlluminaPA' assembly was generated by combining the Sanger PE and 454 SE read collections with preassembled (PA) contigs generated from Illumina PE reads with Velvet. The 'Sanger-454-IlluminaDA' assembly was generated by combining the Sanger PE and 454 SE read collections with Illumina PE reads (DA = direct assembly).

**Figure 2:**
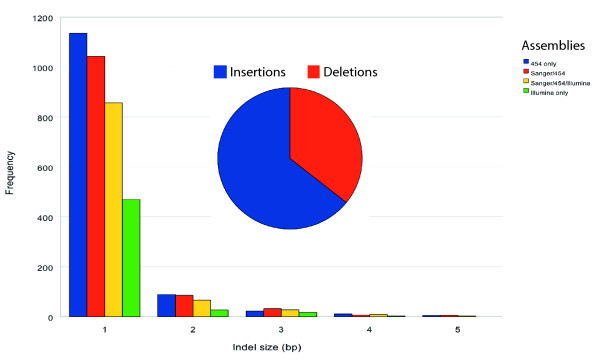
Consensus sequence quality. The proportion of 454 read data within the total read collection affected the number of small insertions and deletions (indels) based on analysis of 7,169 unique EST-to-genome alignments. The relative proportions of insertions (blue) and deletions (orange) in the assembly sequence are shown in the inset pie chart. Assemblies are described in Tables 1 and 2; those including 454 read data were assembled with Forge; the Illumina-only assembly was generated with Velvet.

Comparing indels across assemblies indicated that the rate at which small (1 to 5 bp) insertions or deletions appeared in the assembled consensus sequence depended on the fraction of 454 data in the assembly (Figure 2). When we inspected the frequency of each base that was inserted or deleted across all assemblies that used 454 read data, the pattern was consistently A>T>C>G, while Velvet assemblies of Illumina reads produced a C>A>T>G indel pattern where A, C, G, and T represent indel frequencies for their corresponding bases. To assess whether these small insertions and deletions could disrupt the phasing of the assembled genome sequence (that is, the periodicity of nucleotide sequences within the assembly relative to *cis *factors), we examined the predicted protein collections from each of these assemblies. Average predicted protein sequences contained 401.1 versus 527.0 amino acids in assemblies that used only 454 or only Illumina data, respectively. Although this difference could be the result of an increased contig N50 length in the Illumina based assembly (Tables 1 and 2), we observed that, in the NCBI non-redundant database [8], the fraction of predicted protein sequences with at least one significantly similar sequence was 60% for the 454-only assembly but 70% for the Illumina-only assembly. This suggests that the shorter average protein lengths in assemblies with greater ratios of 454 reads were due to spurious peptide sequences and not contig end truncations. Assemblies that used 454 read data achieved greater amounts of total assembled DNA, including relatively more sequence annotated with repetitive elements, despite shorter contig N50 values; the 454 assembly and the Sanger-454-Illumina assembly were annotated with approximately equal numbers of repetitive elements, while the Velvet assembly had approximately half as many annotations. Because the 454 assemblies also had acceptably low EST-detectable misassembly rates, we concluded that a strategy that combined all three read types would be optimal. We assessed validating our assembly methodology using simulation, but found that the results did not accurately reflect the outcomes of working with real read data. This was likely due to the difficulty of accurately modelling read-specific sequence quality and errors (results not shown).

### Optimizing Sanger/454 assemblies using 454 read filtering

Filtering 454 SE reads for no-calls, length and sequence complexity incrementally improved the overall quality of the *de novo *assembled *Gc *genome sequence relative to a manually finished sequence, which we will refer to as *GC*gb1 (see Materials and methods for a description). For 454 SE reads, no-call filtering removed 95,833 (3%) reads, and length filtering further removed 141 (0.009%) GS20 reads and 3,583 (0.2%) GS-FLX reads. Applying these filtering strategies reduced both the contig and scaffold N50s, suggesting that when a hybrid assembly includes relatively low 454 SE sequence coverage, filtering reads by no-calls and length may be overly aggressive. However, for our strategy of assembling Sanger PE and 454 SE read data around high-coverage Illumina read data, the two filtering steps were worthwhile; applied together, they improved the integration of the different sequence types and reduced the number of chimeric contig ends by 20% (see Supplementary section 1 in Additional data file 1).

Low complexity regions (that is, genome sequences with a simple repetitive composition) are expected features for a filamentous fungus. We found that reads containing such sequences were associated with misassemblies (data not shown). Using DUST [9] we filtered 522 of the Sanger reads and 3,889 of the 454 reads containing such repetitive composition. Filtering 454 and Sanger reads for low complexity sequences marginally affected contig and scaffold N50; however, it reduced the number of scaffolds containing gaps from 685 to 666, and decreased the number of irresolvable split EST alignments by 7. Given this, we removed reads containing low complexity sequence from the draft assemblies. We intend to resolve such regions in the finishing stage of the sequencing project, using tools and resources that are better suited for such genomic elements.

### Improving assemblies with Illumina PE reads by trimming and filtering

Given the promising initial assembly of the Illumina PE read data, we assessed trimming and filtering as a means to improve the Velvet assembly accuracy. Beginning with the 82.6 M, 42-bp PE reads, we discarded 1.1 M reads containing no-call bases and 1.9 M shadow reads (described in Materials and methods). To optimize the Velvet assembly, we used alignments with our preliminary 454 and Sanger sequence assembly to determine trimming and quality read length (QRL; described in Materials and methods) filtering parameters for removing low quality bases from reads (Supplemental section 2 and Figure S4A in Additional data file 1).

As determined by EST alignments and alignments to *GC*gb1, trimming and filtering improved the accuracy while only marginally reducing the total length of DNA assembled; however, more aggressive read trimming and filtering substantially reduced the contig N50s in Velvet assemblies (Table 1). Trimming Illumina reads from 42 bp to 38 bp (T38) and then to 36 bp (T36) reduced the assembly N50 to 10.7 kb and 2.9 kb, respectively. For the T36 assembly, trimming reduced the total amount of assembled sequence and the number of complete EST-to-assembly alignments, while also reducing the number of EST-detectable assembly errors from 29 to 11 (Table 1). Trimming Illumina reads also reduced the effective level of coverage, which likely explains why the N50 and complete EST-to-genome alignments were reduced. Given this, we assessed whether the improvements in EST-detectable assembly errors could also have resulted from arbitrary read trimming and subsequent shortening of the assembled contig lengths. We tested this by removing 6 bp from the 5' end of each read. In the resulting assembly the N50 and complete EST-to-genome alignment counts were approximately half of the corresponding values for the T36 assembly, and the EST-detectable error rate was five times higher, validating the efficiency of our trimming algorithm.

Filtering low quality data (QRL(Q10) = 28) resulted in an assembly that, relative to the T36 assembly, had a smaller N50 (1,299 bp) but only a marginally lower number of EST-detectable assembly errors. We then tested whether filtering by randomly removing the same number of reads that had been removed by QRL filtering changed the resulting assembly. We found that although random filtering did not substantially change N50, it tripled the number of EST-detectable errors and doubled the number of ESTs with no genome assembly alignment, validating the efficiency of our filtering algorithm.

**Figure 3:**
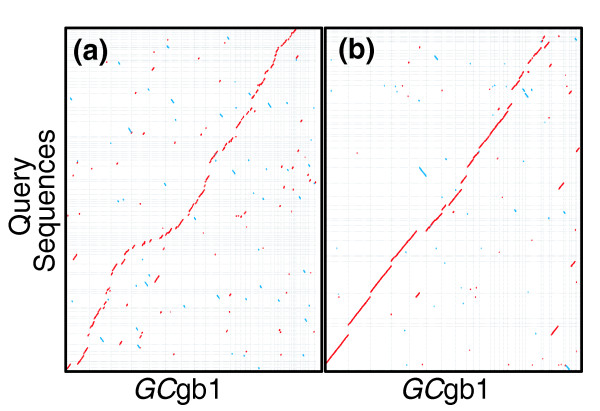
Comparison of Forge Sanger/454/Illumina assemblies against *GC*gb1. Alignments of scaffolds greater that 100 kb - **(a) **'Sanger/454/IlluminaDA' (approximately 24 Mb on 80 scaffolds) and **(b) **'Sanger/454/IlluminaPA' (approximately 28.7 Mb on 46 scaffolds) - on the y-axis against the manually finished genome sequence (*GC*gb1) on the x-axis.

Relative to *GC*gb1, we found that this trimmed and filtered Illumina read collection yielded the most accurate Velvet contigs and that these contigs had approximately 15% fewer chimeric contig ends. Using the approximately 51 M Illumina PE reads resulting from trimming and filtering (approximately 56.5× genome sequence coverage) and the Sanger and 454 data reported above, we attempted two assemblies using a revised version of Forge (v.20090526). We tested: incorporating the Illumina PE data following Velvet preassembly (Sanger-454-IlluminaPA); and incorporating the Illumina PE data directly (Sanger-454-IlluminaDA). EST-to-genome sequence alignments and Illumina PE read alignment cluster analysis showed that the Sanger-454-IlluminaDA genome sequence had a lower misassembly rate than the Sanger-454-IlluminaPA assembly (Table 2). However, alignment to *GC*gb1 suggested that the Sanger-454-IlluminaPA was a more accurate assembly in regards to long range continuity (Figure 3). The Sanger-454-IlluminaDA assembly had greater contig N50 whereas the Sanger-454-IlluminaPA assembly had greater scaffold N50 (Table 1).

### Assessing the final assembly

Assembly Sanger-454-IlluminaPA had 6,314 complete EST alignments and 40 EST-detected assembly errors. The number of scaffolds containing gaps greater than 1 kb, 163, was substantially lower than the 656 in the best assembly achieved without the Illumina PE read data. We assessed the quality of this Forge hybrid assembly using the consistency of the Sanger PE read pairings and 200-bp Illumina PE reads. Adding the Illumina PE read data increased the fraction of consistently-paired Sanger PE reads from 64 to 81% for Sanger-454-IlluminaPA versus the best assembly without Illumina PE read data; for Illumina PE alignment data, the numbers of unpaired reads decreased by 37% and those paired on different scaffolds decreased by 21%, while the number of paired reads on the same scaffold with an appropriate fragment length increased by approximately 1.5 M. The assembly contained 46 scaffolds longer than 100 kb, which represented 88.5% of the total genome sequence. These scaffolds had a G+C content of 53.2%. The 10 largest scaffolds contained 48 gaps with a total length of approximately 181 kb (Figure S5 in Additional data file 1). The longest scaffold was approximately 3.67 Mb and the tenth longest scaffold was approximately 782 kb.

The 454 read coverage and Sanger PE read placements for assembly Sanger-454-IlluminaPA indicate that the distribution of read data was generally uniform across the top ten scaffolds (Figure S5). We noted 12 sequence regions with unexpectedly high read coverage. Preliminary analysis of these sequence regions indicates that, as expected, they were spanned by repetitive elements, primarily transposons. Large gene families with high levels of similarity were also problematic. However, there is no evidence that such genomic elements necessarily ended up in misassemblies; rather, they sometimes caused early contig growth termination by making the collapsed sequence data unavailable to other appropriate genomic regions. Misassemblies primarily occurred when the repeat span was large and fosmid collapses brought incorrect contigs into adjacency during scaffolding. However, these are easily identified and corrected during sequence finishing.

Assessing the final draft assembly using the 200-bp Illumina PE read set highlighted genomic regions with collapsed repetitive elements, low coverage, misassemblies, and adjacent scaffolds. The PE alignment data were plotted by coverage and are shown in Figure S5. Correctly paired read alignments had a mean outside distance of 193 bp and appeared to be evenly distributed across the scaffolds. However, approximately 1,500 anomalous PE read-alignment-clusters (that is, reads with overly stretched gap distances between pairs, unpaired reads or reads paired inappropriately on different scaffolds) highlight that automated rules can be applied to the current draft assembly, and we have implemented a semi-automated system in our finishing pipeline to leverage these data. In *GC*gb1, we have currently resolved > 90% of the anomalous clusters identified in Sanger-454-IlluminaPA. As expected, many (approximately 85%) of the ambiguities that arose during our analysis of PE read clusters occurred at scaffold edges (< 3 kb), suggesting that scaffold growth termination was accurate in this assembly; further, scaffold growth was constrained by read ambiguity rather than by low coverage. Although greater sequencing depth could improve this by allowing better resolution of read overlap alignments, some types of genomic elements will likely continue to cause ambiguity in read overlaps, leading to premature truncation of scaffold growth.

By counting complete gene models for core eukaryotic proteins reported by CEGMA [10], we estimated that we have generated gene models for greater than 94% of the full genome's hypothetical gene model collection. For the preliminary Sanger-454-IlluminaPA gene predictions, the average gene density was approximately 1 gene/3.5 kb, the average gene length was approximately 1.5 kb, the average transcript length was approximately 1.2 kb, and the average transcript G+C content was approximately 58%. Similar values have been reported for other ascomycetes from the order sordariomycetes [11,12]. A detailed description and annotation of the *Gc *genome will be published separately (manuscript in preparation).

### Analysis of Illumina and 454 read data

**Figure 4:**
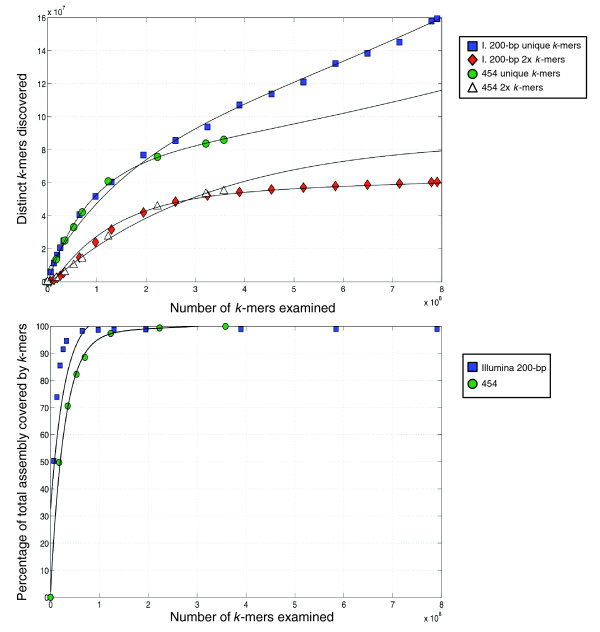
Assessing the discovery of unique read information between the Illumina and 454 platforms. **(a) **Raw reads were processed into overlapping 28-bp *k*-mers, and any *k*-mer that varied from all other *k*-mers by at least 1 bp was accepted as new sequence information. The analysis was done separately for unique *k*-mers and those that occurred at least twice (2× *k*-mers). **(b) **MAQ was then used to map these *k*-mers to the reference genome sequence and the rate at which new coverage was generated was plotted against the number of *k*-mers examined.

We used the manually finished *GC*gb1 assembly to assess the performance of the Illumina and 454 sequencing platforms (Figure 4). We quantified the efficiency of discovering new and useful sequence data, as well as the rate at which the new sequence data covered *GC*gb1. We performed this analysis on all possible read substrings with length 28 bp (termed *k*-mers) generated from the raw reads rather than on the raw reads themselves. Although the rate at which novel *k*-mers were discovered was approximately the same for both technologies at lower numbers of *k*-mers, when we split the analysis of novel *k*-mers into those that appeared at least twice versus once, a greater error rate was observable in the Illumina *k*-mer collection (Figure 4a). Because the 454 read lengths were longer, the unique *k*-mers generated from this read collection overlapped each other more than *k*-mers generated from the Illumina reads. This was inherent in the *k*-mer sampling process and likely explains the slower gain in 454 genome coverage (Figure 4b). Our data were insufficient for systematically assessing library saturation; however, it was apparent that the large number of reads generated for either library captured the entire genome sequence we assembled (Figure 4b). Based on EST-to-genome alignments, approximately 0.6% of the protein coding sequence was missing or ambiguous in *GC*gb1. This could suggest that a portion of the genome remains ambiguous to our assembly methodology or that read data are missing from our sequence set. Given the rapid development of wet lab methodologies, it will be interesting to see whether library saturation remains a challenge for *de novo *genome sequencing.

## Discussion

We sought to rapidly generate a *de novo *genome assembly that supported high quality protein coding gene predictions, wet lab experiments, comparative genomics and sequence finishing for a eukaryotic organism. We used a hybrid approach for sequencing and assembly. We combined Sanger PE, 454 SE and Illumina PE sequence data, and developed an assembly strategy that was adaptable to evolving technologies, tools and methods. Using Forge we generated a draft genome sequence with a length of approximately 32.5 Mb, which had a contig N50 length of approximately 32 kb and a scaffold N50 length of approximately 782 kb. During this work, read lengths and read quality improved for 454 and Illumina platforms; as they changed, we evaluated different ways of processing Illumina sequence reads in order to integrate them into assemblies. We characterized the accuracy of the draft assemblies by aligning ESTs, Illumina PE reads and a manually finished sequence to them.

We chose Forge as the assembler for three reasons. First, it can flexibly integrate different sequencing technologies by automatically adapting alignment parameters for particular read error models. This facilitates using it with evolving sequencing technologies and variable, technology-specific read or contig preprocessing. Second, it is capable of integrating PE information directly into the contig-building and merging processes, making it ideally suited for processing abundant short paired reads. Finally, because it can be run on computer processors running in parallel, it can be applied to the relatively large data sets generated by next-generation platforms. From our initial observations, Forge assemblies were promising as they integrated Illumina PE read data directly, and yielded accurate assemblies with good long range continuity.

Although Forge was designed to accommodate the 454 scoring system, the vendor-supplied quality scores do not indicate the probability that a base is called correctly. While this shortcoming can be addressed by transforming the scores into a Phred-like scale similar to that used for Sanger reads [13], we chose an empirical approach and rejected problematic data [1]. We found that by aggressively applying no-call and length filtering we could improve the overall quality of the assembly, as measured by alignments to the *GC*gb1 sequence, reduced gap sizes and fewer EST-detectable misassemblies. Low complexity filtering was especially useful for the 454 SE read data because, without read pairing information to anchor ambiguous overlaps, accurate read placement appeared difficult to resolve. Although we substantially improved the assemblies using these methods, 454 base calling inaccuracies in the vicinity of homopolymer runs continued to cause phasing problems that affected gene predictions in the assembled consensus sequence. We found that adding Sanger PE reads, Velvet contigs and then Illumina PE reads directly into the assembly progressively improved the consensus sequence by reducing the frequency of these indels. We also found that aligning a collection of Illumina-based assemblies back to the final assembly in a post-processing step accurately identified and resolved these homopolymers.

Given the promising initial assembly of Illumina PE reads, we further assessed how to improve the accuracy of Velvet-assembled contigs. Profiles of read quality and substitution error rate relative to the Sanger/454 preliminary assembly suggested that trimming the 42-bp Illumina reads would improve the assembly accuracy. While trimming reads at position 36 resulted in a lower N50, EST and reference sequence alignments showed that this assembly contained fewer errors; further, these contigs yielded a more accurate Forge assembly than either those with reads trimmed at position 38 or untrimmed. Importantly, adding the Illumina data to Forge assemblies substantially reduced the number of scaffolds and contigs, suggesting that these relatively inexpensive reads contributed additional data and encouraged contig growth and merging.

Forge uses a statistical model of overlap derived from internal simulations to determine the probability that two reads reliably overlap. This probability is systematically lowered or reduced to zero in repetitive regions, forcing Forge to rely on alternative information such as reads with mate pairs anchored in a scaffold, polymorphisms within a repeat family, or the combination of a low probability overlap and read-pair data. An important advance made with Forge during the course of our work was the ability to scale beyond 50 M reads, which enabled the direct integration of Illumina PE read data in a single Forge assembly stage. The increased accuracy of EST-to-genome alignments, Illumina PE read alignments and the significant increase in contig N50 of the resulting assembly likely resulted from the large amount of pairing information introduced by these data. This suggests that when abundant PE information is available, read sequence length is not as important a limitation as anticipated. Currently, one challenge of this assembly method appears to be in balancing out the PE information in the low coverage Sanger data versus the high coverage Illumina data. Although more Fosmid pairs were correctly assigned to the same scaffold in the Sanger-454-IlluminaPA assembly, a greater fraction of the fosmid read pairs had consistent pairing distances in the assembly generated from direct integration of the Illumina PE read data. We also detected fewer inconsistencies in the Sanger-454-IlluminaDA assembly using the Illumina PE alignment strategy. This could have resulted from working directly with the Illumina PE reads in the assembly stage versus working with read substrings (*k*-mers), which is typical in a short read assembler like Velvet. Working with read substrings is an abstraction that does not enforce read integrity onto the contig consensus sequence. For the Illumina PE library reported here, read pairing distances were not distributed normally around the mean, and left hand tailing increased at greater pairing distances (Figure S4B in Additional data file 1). Read pairs with zero gap distance were also noted and could cause occasional sequence deletions in Forge assemblies if not filtered out.

We also noted that although low quality reads did not improve the assembly of genome sequence and so should be filtered out, they remained valuable as PE alignments for assessing and finishing the draft genome sequence. We are assessing the use of additional Illumina PE sequence data to evaluate the quality of the draft genome assembly and to guide finishing. We identified high quality regions in the assembly by calculating the coverage of correctly paired Illumina PE reads, and used scaffold-spanning PE reads to identify possible ambiguities or misassemblies in the consensus sequence. For such assessments, Illumina PE data offer advantages over EST data: the large number of reads provides deeper coverage, and the sequence data include non-transcribed regions, which are typically more difficult to assemble. We were also able to use the PE data to map the boundaries of misassemblies and to link scaffold edges in the consensus sequence. Improved software tools for working with Illumina PE data will likely benefit both the assembly of draft genome sequences and the finishing of these drafts.

In conclusion, we assembled a draft genome sequence for a fungal pathogen using Illumina, 454 and Sanger sequence data. We found that the highest quality assemblies resulted from integrating the read and contig collections in a single round of assembly, using software that could coherently manage the varying read and contig lengths as well as the different error models. Aggressively filtering this high coverage data was an effective strategy for incrementally improving the resulting draft assemblies. We anticipate that the iterative approach that we describe will facilitate using rapidly improving sequencing technologies to generate draft eukaryotic genome sequences.

## Materials and methods

### Library construction and sequencing

*Gc *spores from strain kw1407 [14] were spread onto cellophane overlaid on 1.5% agar containing 1% malt extract in 15-cm petri dishes. The fungal spores were incubated at 22°C in the dark for 8 days, and the mycelia were removed from the cellophane and pooled. DNA was extracted from mycelia following the method of Möller *et al*. [15] but without first lyophilizing the mycelia. For constructing a 40-kb fosmid library, fungal DNA was randomly sheared, then blunt-end repaired and size-selected by electrophoresis on a 1% agarose gel. Recovered DNA was ligated to the pEpiFOS-5 vector (Epicentre Biotechnologies, Madison, WI, USA), mixed with Lambda packaging extract and incubated with host *Escherichia coli *cells. Clones containing inserts were selected and paired-end-sequenced on an ABI 3730xl. For sequencing on the Roche GS20 or GS-FLX sequencers, DNA was prepared using the methods described by Margulies *et al*. [16]. For preparing the approximately 200-bp library on the Illumina GA_ii _sequencer, 5 μg of DNA was sonicated for 10 minutes, alternating 1 minute on and 1 minute off, using a Sonic Dismembrator 550 (Fisher Scientific, Ottawa, Canada). Sonicated DNA was then separated in an 8% PAGE. The library was constructed from the eluted 190- to 210-bp fraction of DNA using Illumina's genomic DNA kit, following their protocol (Illumina, San Diego, CA, USA). Four lanes in a single flow-cell were sequenced to 42 cycles using v.1 sequencing and cleavage reagents. Data were processed using Illumina's GA pipeline (v.0.3.0 beta3).

### Filtering Sanger and 454 reads

For Sanger PE data, we removed reads that had less than 200 bp of continuous sequence with a minimum quality score of Phred 20; 14,522 reads with an average read length of approximately 600 bp remained. We discarded 454 reads that contained uncalled base positions (no-calls), then pooled reads into separate GS20 and GS-FLX sets. After assessing the two read length distributions, we discarded reads whose lengths were either less than 40 bp or longer than 200 bp, or less than 50 bp or longer than 350 bp from the GS20 and GS-FLX sets, respectively, as described by Huse *et al*. [1]. We then applied a low complexity filter to the 454 and Sanger reads using DUST with a 50% threshold [9]. Contamination filtering was performed against a database of bacterial genome sequences. From the initial GS20 read collection approximately 3% of reads were identified with 98% or greater similarity to the genome sequence of *Anaerostipes caccae *and were removed. Lastly, 454 reads were mapped against the Univec database [8] using BLAST to trim and filter library adaptor sequence; 3% of reads were removed and approximately 7.5 Mb of sequence were trimmed from the read collection with no significant difference in the pre- and post-trimming read length (163 bp).

### Assembling Illumina data

Version 7.31 of Velvet is able to generate scaffolded contigs, which results in larger N50 values; however, we were unable to observe scaffolding resulting from our hybrid Sanger/Illumina read assembly. Further, comparing Illumina-only assemblies generated from previous and current Velvet versions to our reference sequence indicated that the contig merging increased the number of assembly errors (data not shown). Given our assembly strategy, the limitations of the Velvet v. 7.31 release indicated that we should continue using Velvet v. 6.04 for our current work.

Because eukaryotic genomes pose an increasing number of ambiguous sequence regions compared with prokaryotes, and because we had generated relatively deep sequence coverage for the 200-bp Illumina library, we used the highest available assembly *k*-mer parameter (hash length) of 31 for all Velvet assemblies reported here. We calculated expected coverage and the coverage cut-off parameters as described in the Velvet documentation.

We applied a simple paired-read analysis to identify chimeric pairs that we believed to be artifacts of library construction and sequencing. We have termed these 'shadow' reads. Briefly, we identified a shadow read pair when a read shares X identical starting bases with its mate, where we tested X equals 6, 8, 10, 12, 14, 16, 20 or 24. We discarded such read pairs with 6-bp or greater shared sequence.

We tested trimming and filtering on the Illumina reads used for assembly and developed a QRL metric using the calibrated Illumina Phred-like quality scores. We calculated a read's QRL as follows. Moving from the 3' towards the 5' end of a read, we used the highest probability score value for each base position to determine a quality score for that base. The maximum possible value for this score is 40. For each read, the QRL was the length between the first and last bases that were above a quality score threshold.

We assessed the Velvet assemblies using four metrics: N50, the scaffold (contig) length for which 50% of the assembled genome is in scaffolds (contigs) that are at least as long as N50; the assembly size, calculated by adding the total length of retained contigs or scaffolds; alignment of the assembly contigs against a manually finished reference sequence; and alignments of ESTs to the assemblies, using a set of 7,169 unique ESTs (each EST was selected as the member with the longest Phred 20 read length from multiple sequence alignments generated by clustering approximately 43 k EST reads) generated in ongoing and previous work [17]. We aligned ESTs using BLASTn with an E-value threshold of 1e-50, and differentiated complete alignments from resolvable and irresolvable partial alignments. Resolvable partial alignments were alignments occurring on a contig edge that could be merged with another partial alignment on a complementary contig edge. Irresolvable partial alignments were alignments in which the partial EST alignments were isolated in the interior sequence region of a contig such that it was not possible to join the complementary alignments. For identifying small insertions and deletions from the same BLAST report, a custom PERL script was used to parse the alignment data; insertions were identified as gaps in the EST query alignment, deletions were identified from gaps in the target side of the alignment. Several of these contig assemblies were then tested in Forge and further assessed using the methodology described below.

Velvet assemblies took approximately 3 hours on a server with two 2.2 GHz dual-core AMD Opteron 275 processors and 8 GB of RAM. Velvet assemblies handled by Forge were assigned base quality scores as uniform PHRED 20 at each position.

### Forge hybrid assembler and genome assembly analysis

The Forge output is a consensus sequence with quality scores and a complete multiple sequence alignment for all reads, with locations in a tabular format that can be converted into the Consed 'ace' file format [18].

We assessed scaffold qualities using the 42-bp PE reads rejected by the filtering process described above in assembling the draft genome sequence. We aligned the reads to the draft assemblies using MAQ [19] in paired-end mode. We processed the output and identified PE relationships using custom PERL scripts and the Vancouver Short Read Analysis Package [20]. We separated the aligned reads into three subsets: PE reads that were correctly spaced and oriented and aligned on the same scaffold; PE reads that were aligned on separate scaffolds; and unpaired read alignments. We used clusters of read-alignment pairs to identify pairs that could be used to merge scaffolds and to identify low quality assembly regions. The first type had a read cluster located at a scaffold edge and a mate-pair cluster located on a complementary scaffold edge. In the second type the complementary cluster was located in the interior scaffold sequence region such that the complementary clusters could not be joined. Because PE read mates can be incorrectly paired in the Illumina flowcell image analysis pipeline, and base-calling errors or low-complexity sequences can result in read placement errors by MAQ, we required cluster sizes of at least 10 before using a cluster to mark a potential scaffold merge or to identify a low-quality region.

As described above, the EST collection was aligned to the Forge assemblies for quality control and alignments were generated against the manually finished genome sequence using nucmer within the MUMmer package with the seed cluster parameter (-c) set to 750. Read coverage, repeat data and quality data were then combined and visualized using Circos [21]. RepeatMasker [22]was used for preliminary filtering of repetitive elements against repbase (v.14) with the species parameter set to 'fungi/metazoa group' prior to gene prediction. Gene prediction was done using Augustus [23]. The Forge hybrid assemblies were generated using the following settings: a genome size estimate of 35 Mb and a hash table size of 80 M for assemblies generated from Sanger/454 read data only or those that included preassembled Illumina PE read data and 260 M for the assembly with direct integration of the Illumina PE read data. The Forge assemblies took 10 to 84 hours on a Linux server cluster using 40 nodes ranging from dual 2.0 GHz processors with 2 GB of RAM to quad-core 2.6 GHz processors with 16 GB of RAM.

### Generating the *GC*gb1 genome sequence

We generated a reference genome sequence and used it for *de novo *assembly verification by using the methodology described above, we added 10,000 additional Sanger fosmid PE reads and approximately 7.6 M, 50 bp Illumina PE reads (see Supplementary section 3 in Additional data file 1). After assembling these data with Forge and applying manual editing, primer walking and other standard finishing techniques; the largest and tenth largest contigs of the resulting genome sequence were 2.33 and 0.68 Mb long, respectively. The largest scaffold was approximately 2.9 Mb and the scaffold N50 was approximately 950 kb. Eighty five percent of the genome sequence was contained within the top 29 scaffolds.

### Data access

Raw read data are available through NCBI genome project ID 39847: fosmid PE Sanger reads (see Additional data file 2 for a complete list of accessions); SE 454 reads [SRA:SRR023307] and [SRA:SRR023517] to [SRA:SRR023533]; 200 bp PE Illumina reads [SRA:SRR018008] to [SRA:SRR018011] and 700 bp PE Illumina reads [SRA:SRR018012]. Assemblies have also been deposited at NCBI: Sanger-454-IlluminaPA [DDBJ/EMBL/GenBank:ACXQ00000000]; Sanger-454-IlluminaDA [DDBJ/EMBL/GenBank:ACYC00000000].

## Abbreviations

EST: expressed sequence tag; GA: Genome Analyzer; *Gc*: *Grosmannia clavigera*; indel: insertion or deletion; NCBI: National Center for Biotechnology Information; PE: paired-end; QRL: quality read length; SE: single-end.

## Authors' contributions

CB, RCH, SJMJ, MAM and JB conceived of the project. SD, NYL, RAH and SJMJ designed the analysis. Sanger sequencing was carried out under the direction of RAH. 454 sequencing was carried out under the direction of EM. Illumina sequencing was carried out under the direction of MH. Forge was developed by DP. Assemblies were performed by SD, NYL, MS, SKC and DP. Data analysis was performed by SD, SKC, TRD and NYL under the direction of IB. The manuscript was prepared by SD, CB, JB and SJMJ with assistance from GR. All authors have read and approved the final version of the manuscript.

## Additional data files

The following additional data are available with the online version of this paper: a PDF file including Supplementary sections 1 to 3, including supplementary Figures S1 to S5 (Additional data file 1); NCBI accession numbers for the trace data used in this study (Additional data file 2).

## Supplementary Material

Additional data file 1 Supplementary sections 1: additional explanation for the 454 read filtering and alignments of the 454 pre- and post- filtered Forge assemblies relative to the manually finished *GC*gb1 sequence. Supplementary sections 2: additional explanation and supporting figures for the filtering and trimming of Illumina PE read data. Supplementary sections 3: supporting Figure S5 detailing the read coverage in the final assembly as well as preliminary repeat annotations and highlighting a small number of misassemblies identified with Illumina PE alignment clusters. Click here for file

Additional data file 2 NCBI accession numbers for the trace data used in this study. Click here for file

## References

[B1] Huse SM, Huber JA, Morrison HG, Sogin ML, Welch DM (2007). Accuracy and quality of massively-parallel DNA pyrosequencing.. Genome Biol.

[B2] Butler J, MacCallum I, Kleber M, Shlyakhter IA, Belmonte MK, Lander ES, Nusbaum C, Jaffe DB (2008). ALLPATHS: *De novo *assembly of whole-genome shotgun microreads.. Genome Res.

[B3] Warren R, Sutton G, Jones S, Holt R (2007). Assembling millions of short DNA sequences using SSAKE.. Bioinformatics.

[B4] Zerbino D, Birney E (2008). Velvet: Algorithms for *de novo *short read assembly using de Bruijn graphs.. Genome Res.

[B5] Simpson J, Wong K, Jackman S, Schein J, Jones SJM, Birol I (2009). ABySS: A parallel assembler for short read sequence data.. Genome Res.

[B6] Forge Genome Assembler. http://sourceforge.net/projects/forge/.

[B7] Pipeline Scripts. ftp://ftp.bcgsc.ca/supplementary/Grosmannia_clavigera/tools/.

[B8] NCBI. http://www.ncbi.nlm.nih.gov.

[B9] DUST. ftp://ftp.ncbi.nlm.nih.gov/pub/tatusov/dust/.

[B10] Parra G, Bradnam K, Korf I (2007). CEGMA: a pipeline to accurately annotate core genes in eukaryotic genomes.. Bioinformatics.

[B11] Galagan JE, Calvo SE, Borkovich KA, Selker EU, Read ND, Jaffe D, FitzHugh W, Ma LJ, Smirnov S, Purcell S, Rehman B, Elkins T, Engels R, Wang S, Nielsen CB, Butler J, Endrizzi M, Qui D, Ianakiev P, Bell-Pedersen D, Nelson MA, Werner-Washburne M, Selitrennikoff CP, Kinsey JA, Braun EL, Zelter A, Schulte U, Kothe GO, Jedd G, Mewes W (2003). The genome sequence of the filamentous fungus *Neurospora crassa*.. Nature.

[B12] Dean RA, Talbot NJ, Ebbole D, Farman ML, Mitchell TK, Orbach MJ, Thon M, Kulkarni R, Xu JR, Pan H, Read ND, Lee YH, Carbone I, Brown D, Oh YY, Donofrio N, Jeong JS, Soanes DM, Djonovic S, Kolomiets E, Rehmeyer C, Li W, Harding M, Kim S, Lebrun MH, Bohnert H, Coughlan S, Butler J, Calvo S, Ma LJ (2005). The genome sequence of the rice blast fungus *Magnaporthe grisea*.. Nature.

[B13] Brockman W, Alvarez P, Young S, Garber M, Giannoukos G, Lee WL, Russ C, Lander ES, Nusbaum C, Jaffe DB (2008). Quality scores and SNP detection in sequencing-by-synthesis systems.. Genome Res.

[B14] Lee S, Kim J, Breuil C (2006). Pathogenicity of *Leptographium longiclavatum *associated with *Dendroctonus ponderosae *to *Pinus contorta*.. Can J Forest Res.

[B15] Möller EM, Bahnweg G, Sandermann H, Geiger HH (1992). A simple and efficient protocol for isolation of high molecular weight DNA from filamentous fungi, fruit bodies, and infected plant tissues.. Nucleic Acids Res.

[B16] Margulies M, Egholm M, Altman WE, Attiya S, Bader JS, Bemben LA, Berka J, Braverman MS, Chen YJ, Chen Z, Dewell SB, Du L, Fierro JM, Gomes XV, Godwin BC, He W, Helgesen S, Ho CH, Irzyk GP, Jando SC, Alenquer ML, Jarvie TP, Jirage KB, Kim JB, Knight JR, Lanza JR, Leamon JH, Lefkowitz SM, Lei M, Li J (2005). Genome sequencing in microfabricated high-density picolitre reactors.. Nature.

[B17] DiGuistini S, Ralph SG, Lim YW, Holt R, Jones S, Bolhmann J, Breuil C (2007). Generation and annotation of lodgepole pine and oleoresin-induced expressed sequences from the blue-stain fungus *Ophiostoma clavigerum*, a Mountain Pine Beetle-associated pathogen.. FEMS Microbiol Lett.

[B18] Gordon D, Abajian C, Green P (1998). Consed: a graphical tool for sequence finishing.. Genome Res.

[B19] Li H, Ruan J, Durbin R (2008). Mapping short DNA sequencing reads and calling variants using mapping quality scores.. Genome Res.

[B20] Fejes A, Robertson G, Bilenky M, Varhol R, Bainbridge M, Jones SJ (2008). FindPeaks 3.1: a tool for identifying areas of enrichment from massively parallel short-read sequencing technology.. Bioinformatics.

[B21] Krzywinski M, Schein J, Birol I, Connors J, Gascoyne R, Horsman D, Jones SJ, Marra MA (2009). Circos: an information aesthetic for comparative genomics.. Genome Res.

[B22] RepeatMasker. http://www.repeatmasker.org/.

[B23] Stanke M, Schöffmann O, Morgenstern B, Waack S (2006). Gene prediction in eukaryotes with a generalized hidden Markov model that uses hints from external sources.. BMC Bioinformatics.

[B24] The Tria Project. http://www.thetriaproject.ca/index.php.

