# Supplementary material for: De novo genome sequence assembly of a filamentous fungus using Sanger, 454 and Illumina sequence data
Source: Genome Biol. 2009 Sep 11;10(9):R94. doi: 10.1186/gb-2009-10-9-r94 (PMC2768983; doi:10.1186/gb-2009-10-9-r94)

Supplementary section 1: 454 SE read filtering

Following methods developed by Huse et. al. [1] we used an empirical system for removing low quality 454 SE read data. In the Sanger/454 series of assemblies filtering reads by no-calls or length increased the number of consistently paired Sanger reads on the same scaffold by 54 and 27, and decreased the number of EST-detectable misassemblies by 9 and 3 respectively. Additionally, length filtering reads reduced the 454 associated indel rate by ~10%. Although applying both filtering methods reduced the number of EST-detectable misassemblies by 4, it also decreased the number of consistently paired Sanger reads on the same scaffold by 72, compared with the unfiltered 454 read assembly. As the 454 read coverage dropped, after applying our read filtering steps, the number of contigs assembled with Sanger reads decreased because removed 454 read data prevented overlap alignments with Sanger reads and subsequent integration. However, the higher quality data improved the quality of the assembled contigs, more accurately placing the Sanger PE reads leading to improved assembly scaffolding (Figure S2). This became more critical as the quantity and variety of the read data increased in later assemblies.

Figure S1. Filtering by no-calls, length and complexity improved Sanger/454 hybrid assemblies.

A. Alignment of the top 25 scaffolds from the assembly generated using filtered and trimmed Sanger reads and unprocessed 454 reads (Table 1) against the manually finished reference genome sequence. The top 25 scaffolds account for X bp of sequence, and the range from largest to smallest was X to X respectively. The contig N50 for this draft assembly was X kb. **B.** As for (A), but for an assembly generated with the 454 read collection after filtering for no-calls, length and complexity. The top 25 scaffolds accounted for X bp of sequence, and the range from largest to smallest is X to X respectively. The contig N50 for this draft assembly was X.

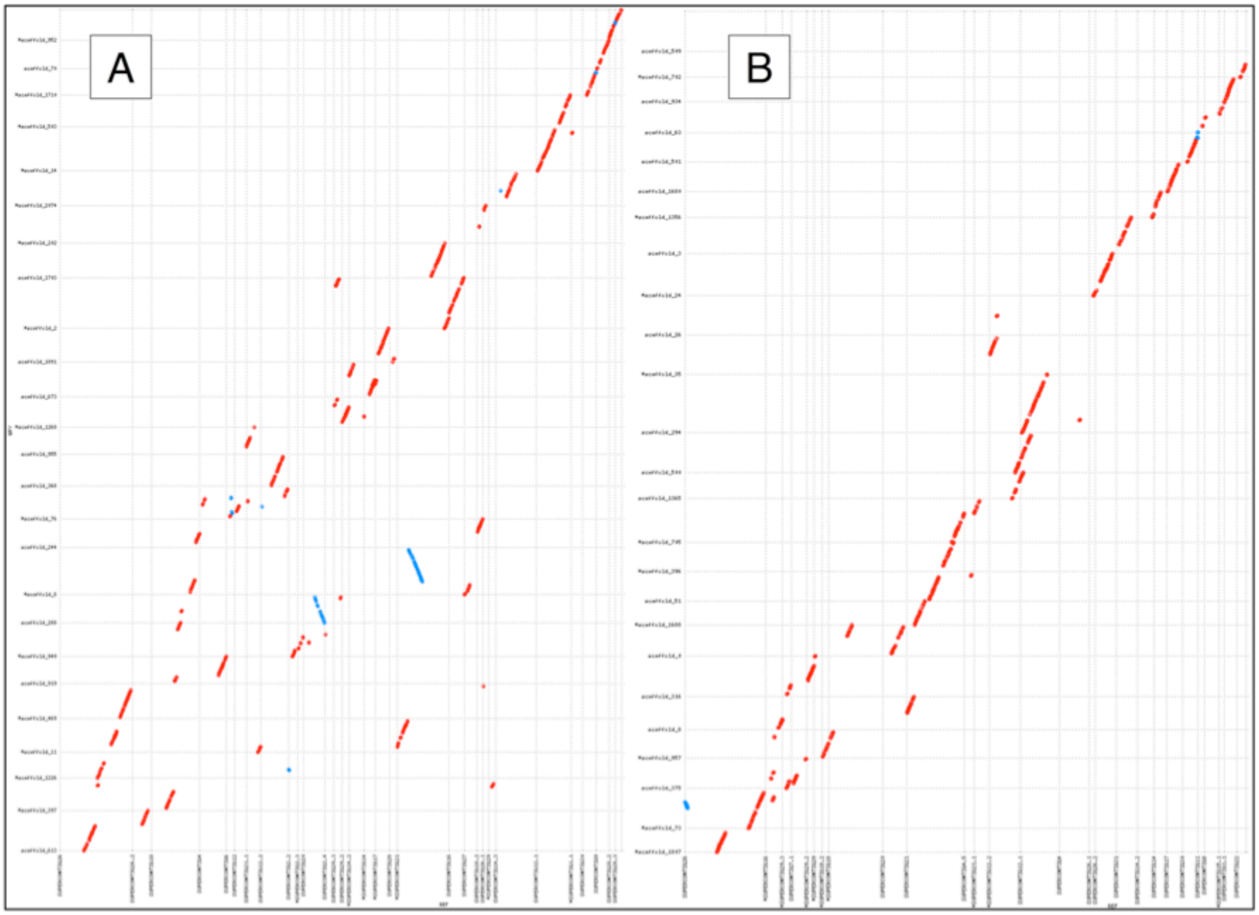

Supplementary section 2: Trimming and filtering Illumina PE reads

To remove low quality Illumina PE read data we profiled average quality scores (Q) (Figure S3) and substitution rates along reads (Figure S4), we used MAQ [15] with Illumina's GA_{ii} calibrated quality scores to align the reads to an intermediate assembly that we had generated with Forge using only the filtered Sanger and 454 read data. From these read mappings, we calculated the average rate of substitution at each read position. For these calculations, we used reads that passed Illumina's default filtering (chastity ≥ 0.6) and had up to three mismatches in total (seed length = 28 bp; seed alignment ≤ 2 mismatches). Substitution rates were evaluated against the fraction of reads within three quality score ranges at the same read position. For the ~200-bp library (Figure S4A) quality scores decreased at a moderate rate along a read, then decreased sharply between 31/32 and then again at read positions 35/36. These sharp transitions, which are likely artefacts of the Illumina base calling pipeline, did suggest positions where trimming might improve the Velvet assembly by removing low quality bases at the 3' ends of reads. Both the substitution rate and the rate of lowest quality base calls increased gradually towards read ends. Low quality reads (quality score (Q) ≤ 10) had a higher rate of base substitutions relative to the reference assembly (Figure S5A). This rate increased by ~7% between read positions 28 and 29, likely an artefact of the MAQ 28-bp alignment seed length, and then increased steadily towards the read end. Empirically we found that filtering reads containing low quality base calls beyond position 28 (*i.e.* QRL(Q10)=28) improved the quality of the Velvet assembly.

For the Illumina ~650-bp library used to generate the reference assembly we started with a

collection of ~24.2 M 50 bp PE reads, shadow filtering removed ~2.9 M reads and purity filtering removed ~7.3 M reads. Based on the initial read mapping results we observed a large number of reads that mapped with a zero gap distance and required filtering. We removed ~2.3 M reads with zero gap distance and then applied a QRL(Q10)=42 filter based on the results plotted in Figures S(3,4 and 5)B and removed ~4.1 M reads.

Figure S2. Assessing the quality of Illumina PE read data by average quality score (Q) at each read position

An average read quality score was calculated from quality scores (Q) extracted from the Illumina base-calling pipeline's 'export' file and plotted for each read position.

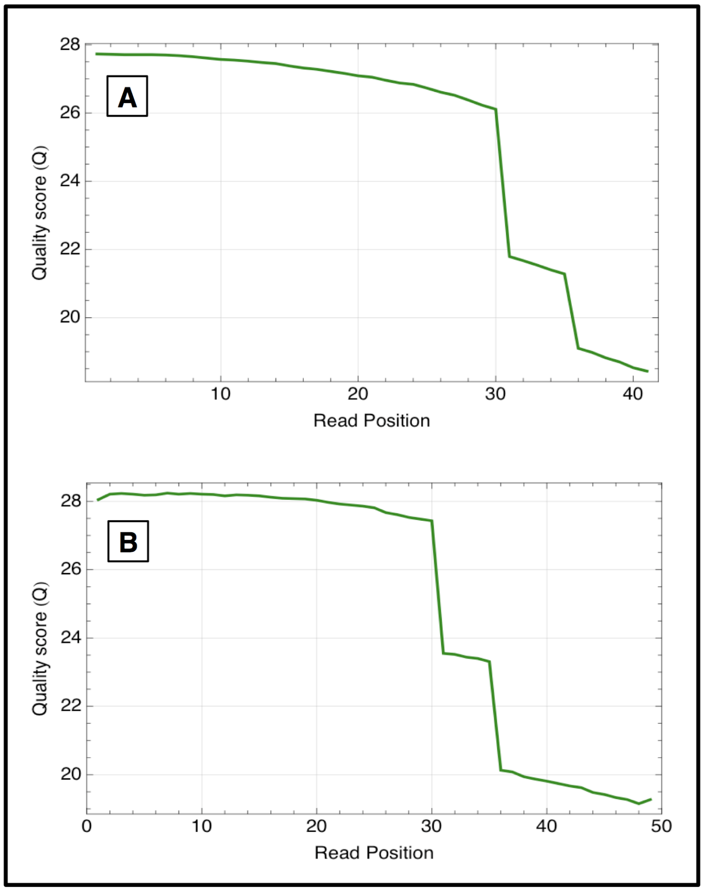

Figure S3. Assessing the quality of Illumina PE read data with profiles of substitution errors and read quality scores.
Read quality scores were extracted from the Illumina base-calling pipeline's 'export' file. Base substitution sequencing errors were estimated as follows. MAQ was used to align reads to the preliminary Sanger/454 read assembly. From these read alignments, mismatches were tabulated and compared with the Illumina quality scores as a function of read position. The inset shows the library fragment length distribution determined from distances between mapped read pairs (measured from 5' to 3' of the mapped reads).

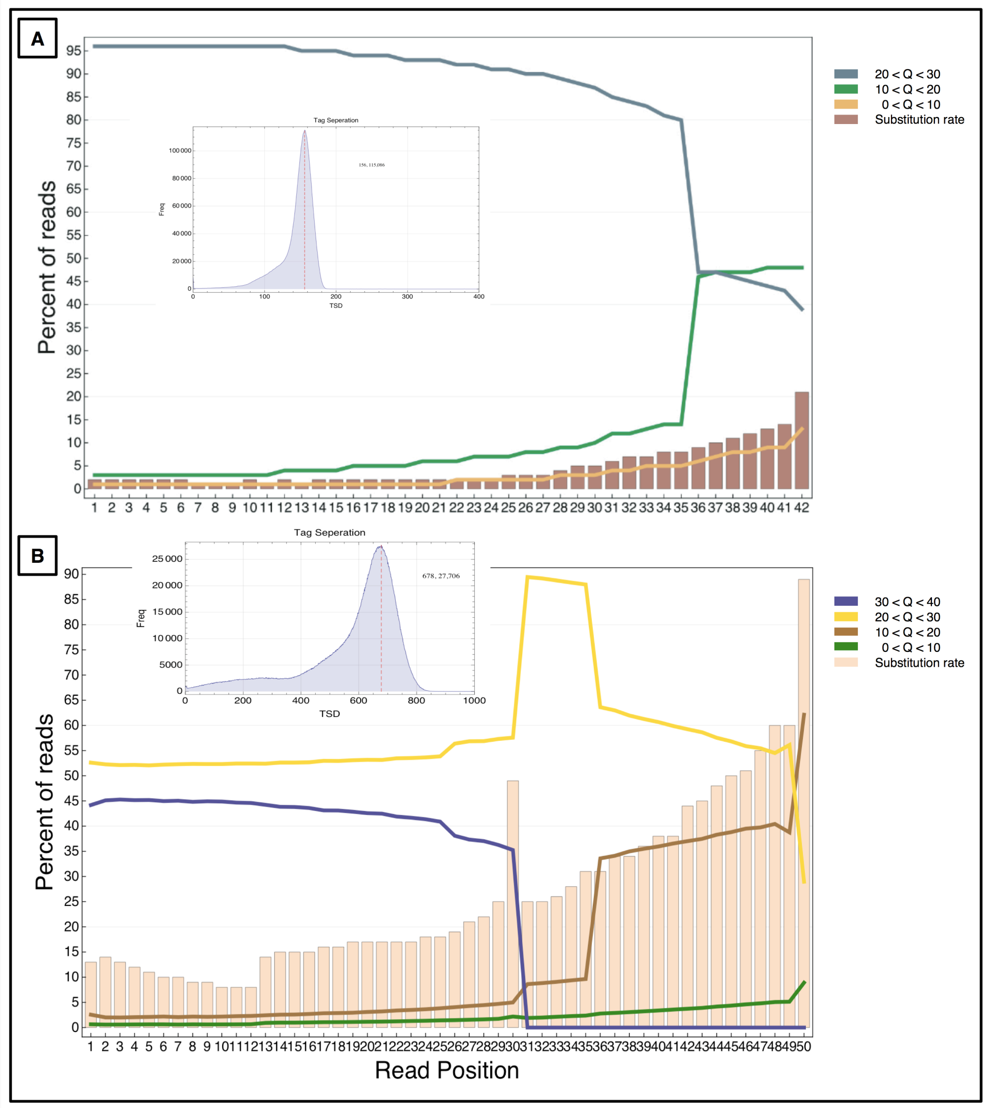

Figure S4. Fraction of reads with substitutions by read position

Using the MAQ data reported in Figure S4, we plotted the fraction of reads within each quality bin reported to have a sequence variation compared with the reference sequence.

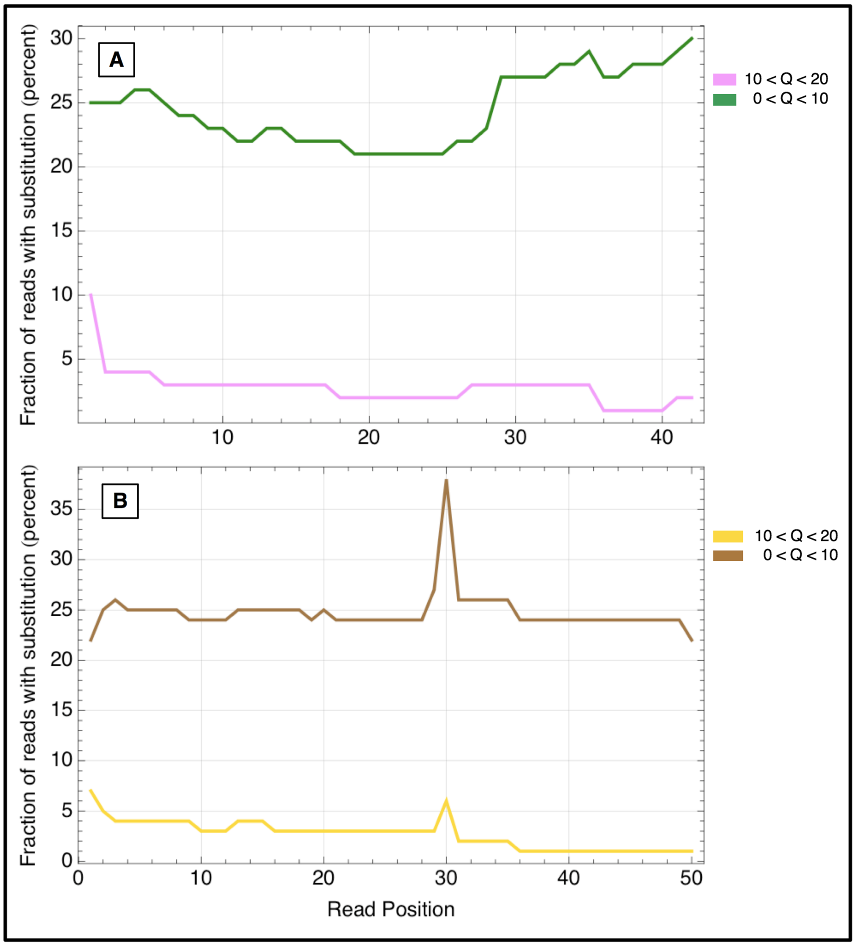

Supplementary section 3: Assessing Forge assemblies that integrate Illumina PE read data directly.

Figure S5. Overview of the largest 10 scaffolds from the best Forge assembly.
From the outside towards the inside of the Circos plot (mkweb.bcgsc.ca/circos): Bold numbering indicates the scaffold IDs. 2) solid black lines represent scaffolds, and are marked by a kb length scale. 3) green tiles indicate density of Sanger PE reads placed by Forge. 4) orange tiles indicate density of the preassembled Velvet contigs placed by Forge. 5) black plot/orange background indicates 454 read coverage averaged over a 2.5 kb window. 6) black plot/green background indicates the coverage of correctly paired Illumina GA_{ii} data averaged over a 2.5-kb window. 7) black tiles indicate the density of sequences masked by RepeatMasker. 8) arcs indicate pooled Illumina PE alignment clusters spanning different assembly scaffolds; these clusters indicate scaffold misassemblies.

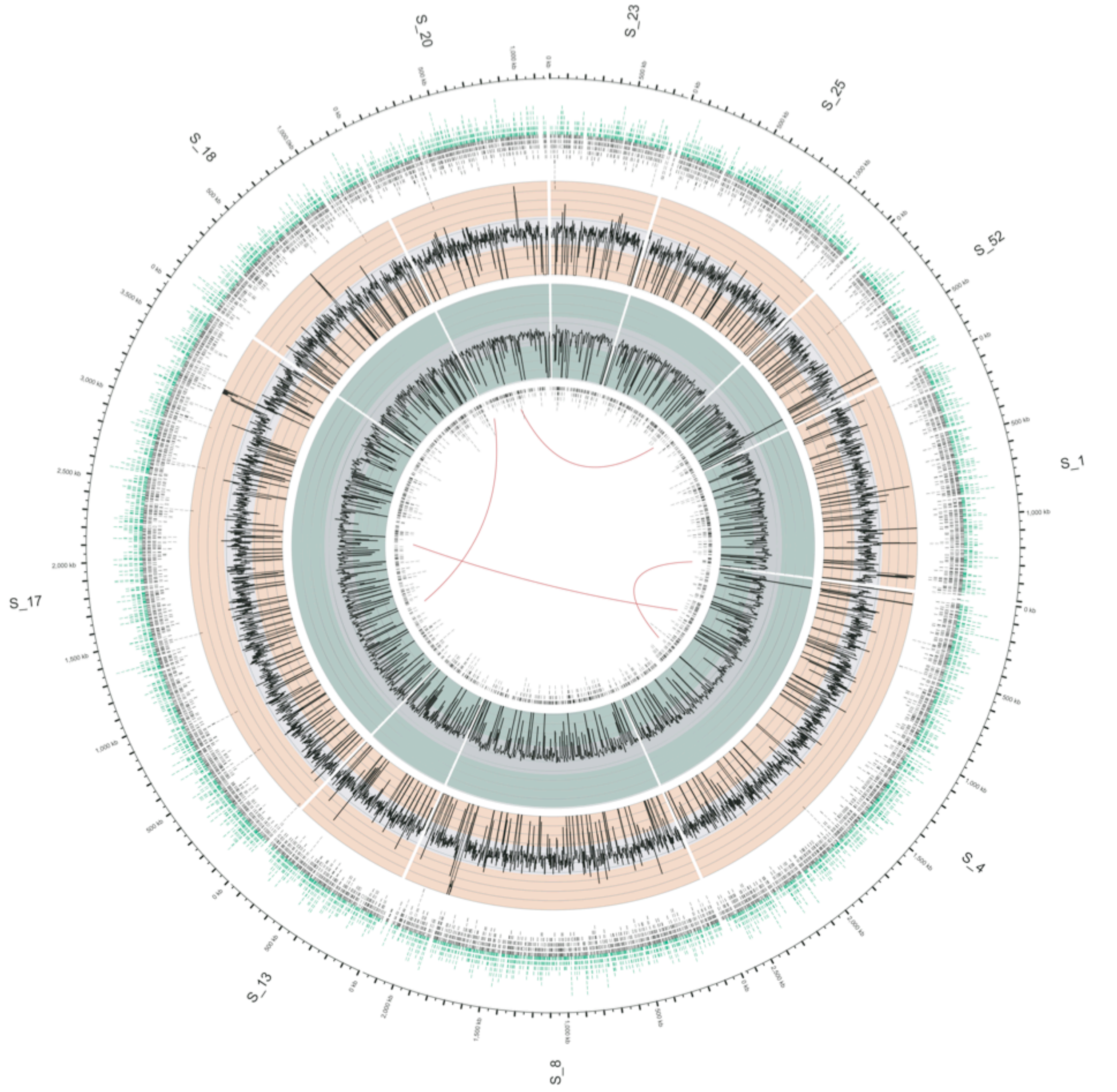

Supplement: Additional data file 1 — Supplementary sections 1: additional explanation for the 454 read filtering and alignments of the 454 pre- and post- filtered Forge assemblies relative to the manually finished GCgb1 sequence. Supplementary sections 2: additional explanation and supporting figures for the filtering and trimming of Illumina PE read data. Supplementary sections 3: supporting Figure S5 detailing the read coverage in the final assembly as well as preliminary repeat annotations and highlighting a small number of misassemblies identified with Illumina PE alignment clusters. [file gb-2009-10-9-r94-S1.pdf]
